# Bioinformatics for Dentistry: A secondary database for the genetics of tooth development

**DOI:** 10.1371/journal.pone.0303628

**Published:** 2024-06-06

**Authors:** Ava K. Chow, Rachel Low, Jerald Yuan, Karen K. Yee, Jaskaranjit Kaur Dhaliwal, Shanice Govia, Nazlee Sharmin

**Affiliations:** School of Dentistry, College of Health Sciences, Faculty of Medicine & Dentistry, University of Alberta, Edmonton, Canada; University of Puthisastra, CAMBODIA

## Abstract

Genes strictly regulate the development of teeth and their surrounding oral structures. Alteration of gene regulation leads to tooth disorders and developmental anomalies in tooth, oral, and facial regions. With the advancement of gene sequencing technology, genomic data is rapidly increasing. However, the large sets of genomic and proteomic data related to tooth development and dental disorders are currently dispersed in many primary databases and literature, making it difficult for users to navigate, extract, study, or analyze. We have curated the scattered genetic data on tooth development and created a knowledgebase called ‘Bioinformatics for Dentistry’ (https://dentalbioinformatics.com/). This database compiles genomic and proteomic data on human tooth development and developmental anomalies and organizes them according to their roles in different stages of tooth development. The database is built by systemically curating relevant data from the National Library of Medicine (NCBI) GenBank, OMIM: Online Mendelian Inheritance in Man, AlphaFold Protein Structure Database, Reactome pathway knowledgebase, Wiki Pathways, and PubMed. The accuracy of the included data was verified from supporting primary literature. Upon data curation and validation, a simple, easy-to-navigate browser interface was created on WordPress version 6.3.2, with PHP version 8.0. The website is hosted in a cloud hosting service to provide fast and reliable data transfer rate. Plugins are used to ensure the browser’s compatibility across different devices. Bioinformatics for Dentistry contains four embedded filters for complex and specific searches and free-text search options for quick and simple searching through the datasets. Bioinformatics for Dentistry is made freely available worldwide, with the hope that this knowledgebase will improve our understanding of the complex genetic regulation of tooth development and will open doors to research initiatives and discoveries. This database will be expanded in the future by incorporating resources and built-in sequence analysis tools, and it will be maintained and updated annually.

## Introduction

Tooth development is a complex process regulated by an intricate network of genes and proteins, that can also be influenced by environmental factors or epigenetic modifications [1]. Recent advancements in molecular signaling have provided insights into the dynamic epithelial-mesenchymal interactions regulating the spatial arrangements and temporal development of teeth [2]. Developmental anomalies of tooth and facial regions and genetic disorders of teeth are often linked to mutations in genes involved in tooth development [3]. Dental and oral disorders account for significant morbidity and healthcare spending each year [4]. In 2016, dental caries and periodontitis were reported as the 11^th^ most prevalent disease worldwide by the Global Burden of Disease [4]. As indicated in the World Health Organization (WHO) Global Oral Health Status Report (2022), oral diseases affect around 3.5 billion people across the world [5]. However, the genomic basis of oral and dental disorders is still largely uncharacterized.

Early histological studies aimed to understand tooth development and eruption from the cross-section of oral tissues were mostly conducted on animal models, as they are not feasible for humans. Most studies on human tooth eruption were longitudinal studies focused on normal and pathological conditions [6]. Many early genomic studies aimed to investigate oral diseases were also based on the comparison between the affected and unaffected individuals and differences between the allelic frequency [7].

With the advancement of gene sequencing technology, sequence data for genes and proteins are increasingly becoming available in primary databases like NCBI, which includes data related to tooth development and developmental anomalies. A large amount of genetic information, including gene sequences, gene loci, protein sequences, protein structures, interactions, and associated mutations, needs to be adequately organized and annotated to better understand the genetic regulation of tooth development and developmental disorders. Tooth development is also an excellent system for studying molecular mechanisms of organogenesis, development, and embryonic morphogenesis [2].

Understanding the genomics of tooth development can improve approaches to risk assessment, hereditary patterns, outcome prediction, and treatment plans [8]. Analyzing sequence variation can identify the increased risk of many conditions like oral cancer, periodontal disease, and cleft lip and palate [9]. With the knowledge of genetic variations, oral health professionals can develop more targeted treatments and personalized preventive measures that can improve treatment outcomes and reduce the risk of side effects [9, 10].

In recent years, genome-wide association studies have identified variations in the human genome to be associated with several dental, oral, and craniofacial traits, including periodontal disease [11], dental caries [12], tooth agenesis [13], orofacial clefts [14] and many more. This new line of research creates opportunities for the dental and oral health community and necessitates educating emerging oral health professionals with the knowledge of genomics, proteomics, and bioinformatics to face the challenges and understand the full potential of genomics and precision health care [10].

Biological databases, which archive large collections of biological data, can be primary, secondary, or hybrid [15]. Primary databases, like GenBank [16] and Protein Databank [17], contain experimentally derived data such as nucleotide sequences, protein sequences, or macromolecular structures. Secondary databases, also known as curated databases or knowledgebases, archive information derived or analyzed from primary databases to meet specific research needs. PROSITE of the Swiss Institute of Bioinformatics [18] is an example of a secondary database consisting of an extensive collection of DNA sequence motifs or patterns. Databases with both primary and secondary nature are called hybrid databases. UniProt [19], a hybrid database, accepts primary sequences derived from peptide sequencing experiments and also provides additional information derived from other primary databases [15].

Genomic and proteomic data related to tooth development and dental disorders are currently dispersed in many primary databases and literature, making it difficult for researchers and students to study or analyze. Some databases that are currently active and dedicated to oral biology include eHOMD which archive information on bacteria in the human mouth; SalivaTecDB, a database of human salivary protein; and the Clinical Genomic Database (CGD), a manually curated database of conditions with known genetic causes (Table 1). CGD reports 202 records in the dental organ system in the manifestation category- ‘Dental.’ To the best of our knowledge, a repository dedicated to the genomics and proteomics of tooth development is not available.

**Table 1:** Currently active biological databases aiming to archive data related to oral biology.

Database related to oral biology		Brief description
eHOMD expanded Human Oral Microbe Database	https://www.homd.org/	eHOMD provides comprehensive curated information on bacteria in the human mouth and aerodigestive tract, including the pharynx, nasal passages, sinuses and esophagus.
SalivaTecDB	http://salivatec.viseu.ucp.pt/salivatec-db/main.php	SalivaTecDB is a database dedicated to the annotation and characterization of proteins, miRNAs and microorganisms present in the oral cavity associated with oral or systemic mechanisms.
Clinical Genomic Database (CGD)	https://research.nhgri.nih.gov/CGD/	Clinical Genomic Database (CGD), a manually curated database of conditions with known genetic causes, focusing on medically significant genetic data with available interventions.

Developing a secondary database with curated data is time and resource-consuming and depends on the availability and accuracy of datasets in the primary databases. However, considering the importance of tooth development and developmental anomalies, we undertook the effort of curating the scattered information related to tooth development and creating a knowledgebase called Bioinformatics for Dentistry (https://dentalbioinformatics.com/). Unlike CGD, the only other database archiving genomic data related to dental disorders, Bioinformatics for Dentistry compiles genomic and proteomic data on human tooth development, categorized based on cellular processes, like enamel formation and tooth eruption. This database is unique, current, and is not a duplication of existing work.

## Materials and methods

### Search and inclusion

Bioinformatics for Dentistry is built by systemically extracting relevant data from multiple sources, including the National Library of Medicine (NCBI) GenBank [16], OMIM: Online Mendelian Inheritance in Man [20], AlphaFold Protein Structure Database [21], Reactome pathway knowledgebase [22], Wiki Pathways [23] and PubMed [24]. For each set of genes that are involved in a stage of tooth development, the following systematic approach was used:

1. GenBank of NCBI was searched using the keywords ‘[Tooth development stage]’ AND ‘Human’ to identify candidate genes involved in a particular stage of tooth development.
2. A search for peer-reviewed primary literature was conducted to verify the involvement of the candidate genes in tooth development through experimental evidence. Genes with no documented function in tooth development were then excluded from the list.
3. For each gene included in the final list, further data collection was conducted to curate information related to chromosomal location, protein-related information, cellular pathways, dental and oral disorders, and reported mutations.

**Fig 1:**
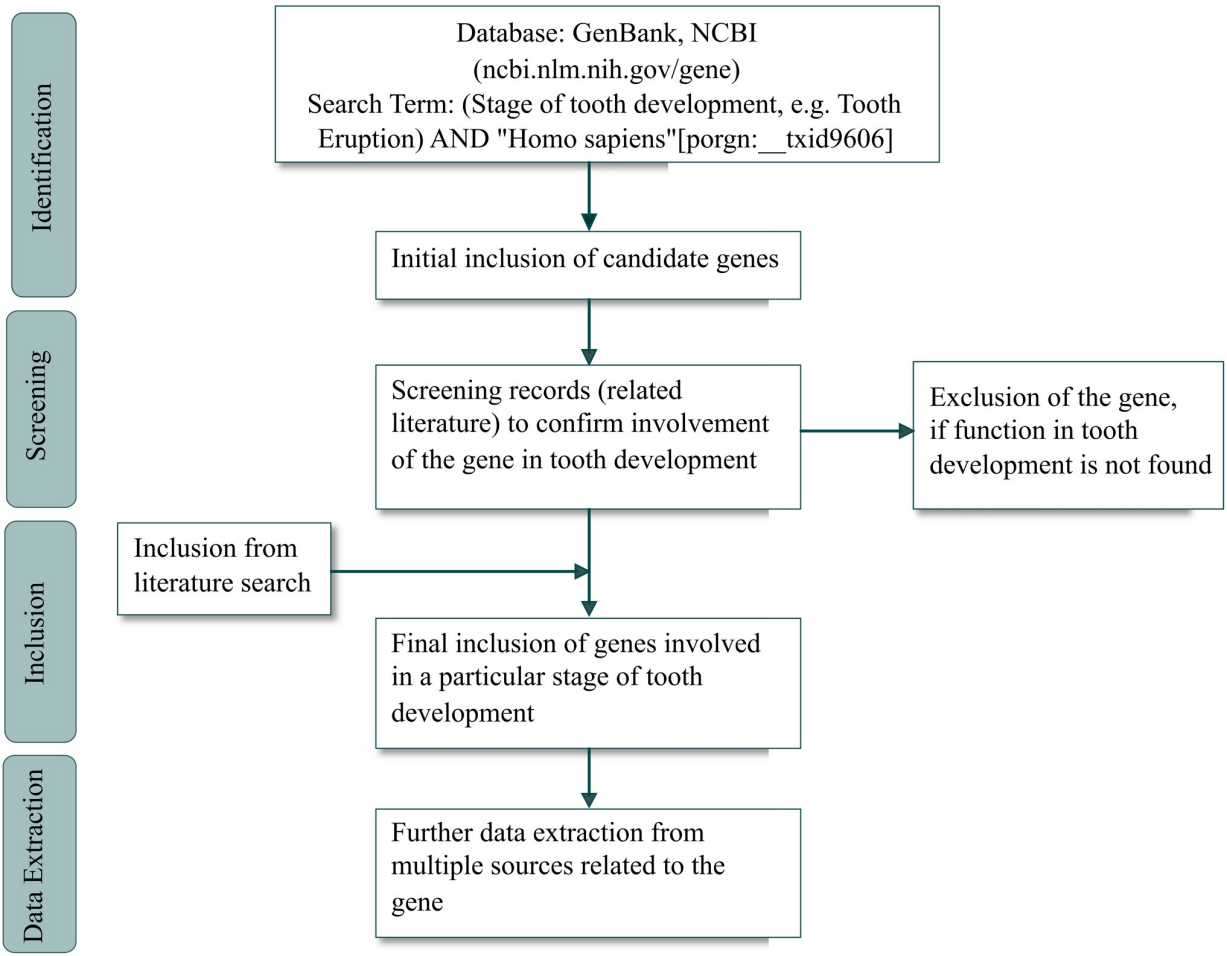
The search strategy for data inclusion in the Bioinformatics for Dentistry.

The detail of the search strategy is shown in Fig 1.

### Data collection

The genomic data was collected and organized according to their roles in different stages of tooth development (cellular process), which include the bud, cap, and bell stages of tooth development, enamel formation, dentin formation, and tooth eruption. The Gene ID, general description, alternative titles (symbols), cytogenetic location, and encoded protein sequence were extracted for each gene. For each protein encoded by the gene, the protein sequence, data for dental and oral disorders, disease-causing mutations, and related literature were further extracted. A brief description of the protein function is also collected from the literature. Attention was paid to maintaining the consistency of terminology. Two pathway databases were searched for the protein to identify signaling networks. Knowledge of three-dimensional (3D) protein structure is essential to studying protein function. However, few proteins involved in tooth and facial development have been fully crystalized or studied in detail. In recent years, advancements in detecting distant homologs, sequence alignment, and loop modeling have contributed to the reliable prediction of protein structure [25]. For each protein in the database, the predicted 3D structures from AlphaFold [21], an artificial intelligence (AI) system developed by DeepMind for predicting 3D protein structure from its amino acid sequence were extracted. In addition, the AI-predicted 3D structure, homology models were developed from the iterative threading assembly refinement (I-TASSER) [26] server for several proteins, which is currently included in the Bioinformatics for Dentistry database as static images. For each sequence input in I-TASSER, five predicted protein structures are generated with corresponding C-scores, TM-scores, and root-mean-square distance (RMSD) values [26]. Higher C-scores represent higher confidence for the predicted protein homology model. TM-score and RMSD are estimated based on C-score and protein length following the correlation observed between these qualities. For the proteins of this database, the best-predicted models were chosen from I-TASSER based on the C-score, TM-score and RMSD. The primary sources of all the data in the database are listed in Table 2.

**Table 2:** Content of the Bioinformatics for Dentistry, with its respective primary sources.

Content of the Database	Sources
***Genes involved in human tooth development*** Bioinformatics for Dentistry archives 132 human genes involved in tooth development. The genes are organized according to the stages of tooth development. For each gene entry, ‘Gene ID’ and link to the primary GenBank page is also included, providing access to the DNA sequence.	GenBank: National Library of Medicine (NCBI) www.ncbi.nlm.nih.gov/gene
***Alternative symbol*, *chromosome number*, *and cytogenetic location*** For each of the 132 entries, all known alternative symbols, chromosome numbers, and specific cytogenetic locations are provided.	OMIM: Online Mendelian Inheritance in Man www.omim.org/
***General description of the gene*** This database section contains a brief general description of the gene and its encoded product.	GenBank: National Library of Medicine (NCBI) www.ncbi.nlm.nih.gov/gene
***Proteins involved in human tooth development*** For each gene entry, information related to the encoded protein is incorporated. Link is provided for users to visit the source-page of the protein from NCBI.	Protein: National Library of Medicine (NCBI) www.ncbi.nlm.nih.gov/protein/
***Protein sequence*** The database archives the sequence of each protein in FASTA format.	Protein: National Library of Medicine (NCBI) www.ncbi.nlm.nih.gov/protein/
***Protein structure*** Knowledge of three-dimensional (3D) protein structure is essential for studying protein function. However, crystalized, complete protein structures related to teeth and oral development are rare. As an alternative to experimentally determined structure, users have access to computationally generated protein models from Bioinformatics for Dentistry database. For each protein listed in the database, uses have access to its interactive 3D protein structure generated from amino acid sequence using artificial intelligence. For many proteins of the database, the users have additional access the static images of the homology models developed by the iterative threading assembly refinement (I-TASSER) server.	AlphaFold: Protein Structure Database https://alphafold.ebi.ac.uk/ I-TASSER: Protein Structure & Function Predictions https://zhanggroup.org/I-TASSER/
***Function of the protein in oral and tooth development*** Users can learn about dental specific function of each protein included in the database. This section contains a brief description and experimental evidence confirming the role of the protein in tooth development.	OMIM: Online Mendelian Inheritance in Man www.omim.org/ PubMed https://pubmed.ncbi.nlm.nih.gov/
***MicroRNA modulators of Tooth development*** Several genes involved in tooth development codes for microRNAs. Users of the database have access to the detail information of these microRNA modulators.	GenBank: National Library of Medicine (NCBI) www.ncbi.nlm.nih.gov/gene
***Cellular Pathways*** For each protein listed in the database, users have access to cellular and metabolic pathways where the protein is known to play roles.	Reactome pathway knowledgebase www.reactome.org/ WikiPathways www.wikipathways.org/
***Dental and Oral Disease*** Bioinformatics for Dentistry achieves the oral and dental diseases linked to the gene entries. Users can get the OMIM ID for each disorder, which will help them to study more from the primary source.	OMIM: Online Mendelian Inheritance in Man www.omim.org/
***Mutation*** For the gene entries, known mutations identified to cause dental and oral diseases are listed in the database.	OMIM: Online Mendelian Inheritance in Man www.omim.org/
***Literature*** Bioinformatics for Dentistry provides access to the primary literature related to protein function, dental disorders, and reported mutations.	PubMed https://pubmed.ncbi.nlm.nih.gov/

### Database architecture and website creation

**Fig 2:**
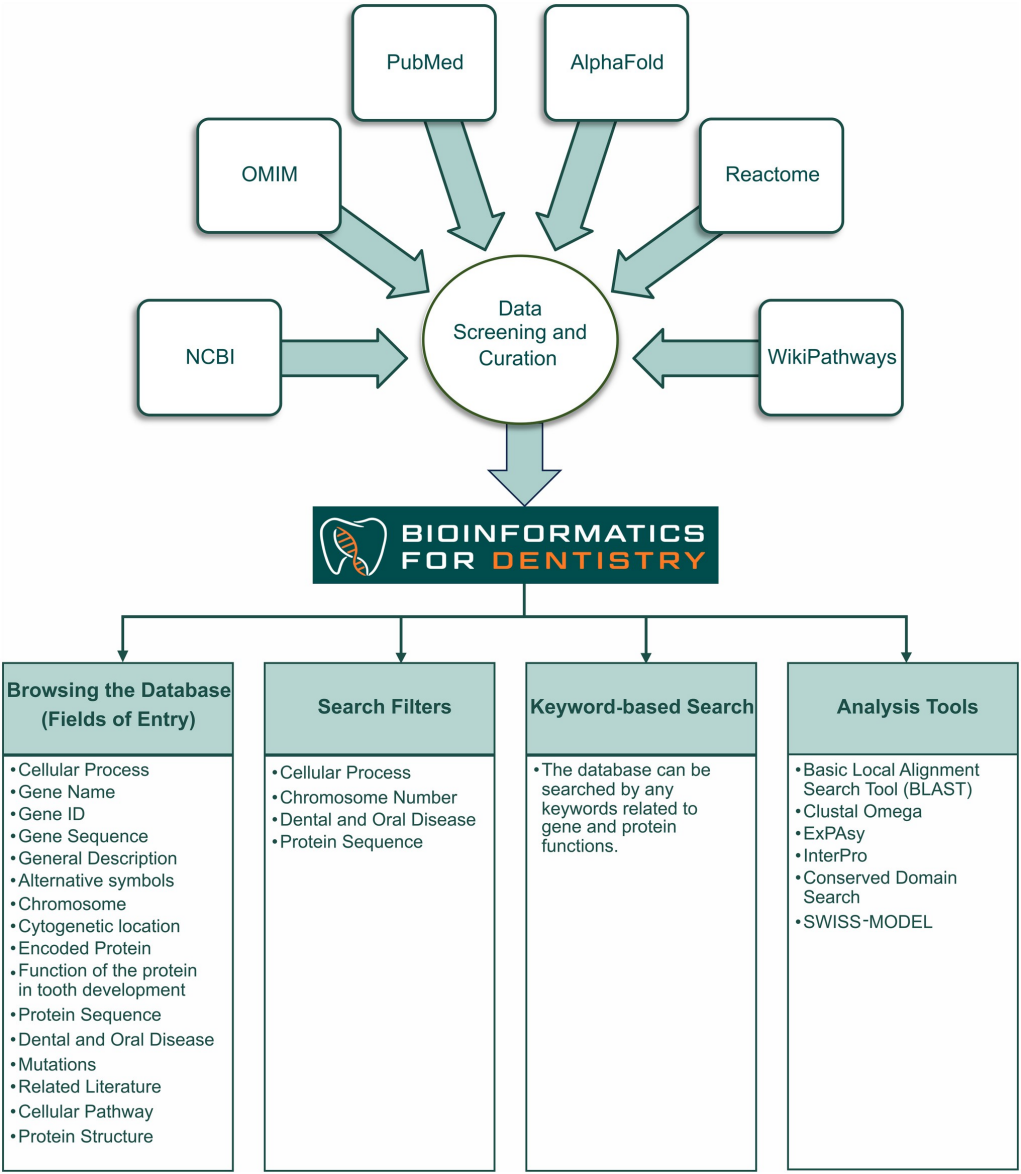
The architecture of the database, Bioinformatics for Dentistry.

The collected data were organized to maximize accessibility in the database. The database architecture is shown in Fig 2. Upon data curation, a user interface was created for ‘Bioinformatics for Dentistry’ on WordPress version 6.3.2, with PHP version 8.0. Additional functions for the website were created using HTML, CSS, and PHP programming languages. The landing page and the auxiliary information pages were created using the Elementor Pro tools on the WordPress graphical user interface. Crocoblock JetEngine and CSS coding were used to create the database template page showing specific information for each structure. By using Jetsmart Filter, the datasets were categorized into different types of structures. PHP was used to build the infrastructure for simple keyword searches and intricate filtering searches.

To ensure compatibility of the database user interface across different devices Elementor and Elementor Pro plugins were used. The Yoast SEO plugin was used for search engine optimization. To maintain the security of the database, SSL (Secure Sockets Layer) certificate, and other necessary security plugins were installed. The website is hosted in a cloud hosting service to ensure a fast and reliable data transfer rate.

This study was reviewed by the University of Alberta Research Ethics Board 2 (Study ID: Pro00107559). As there are no active participants in this work and all information was taken from publicly accessible sources, the requirement for consent was not applicable.

## Results and discussion

### Content of the database

**Fig 3:**
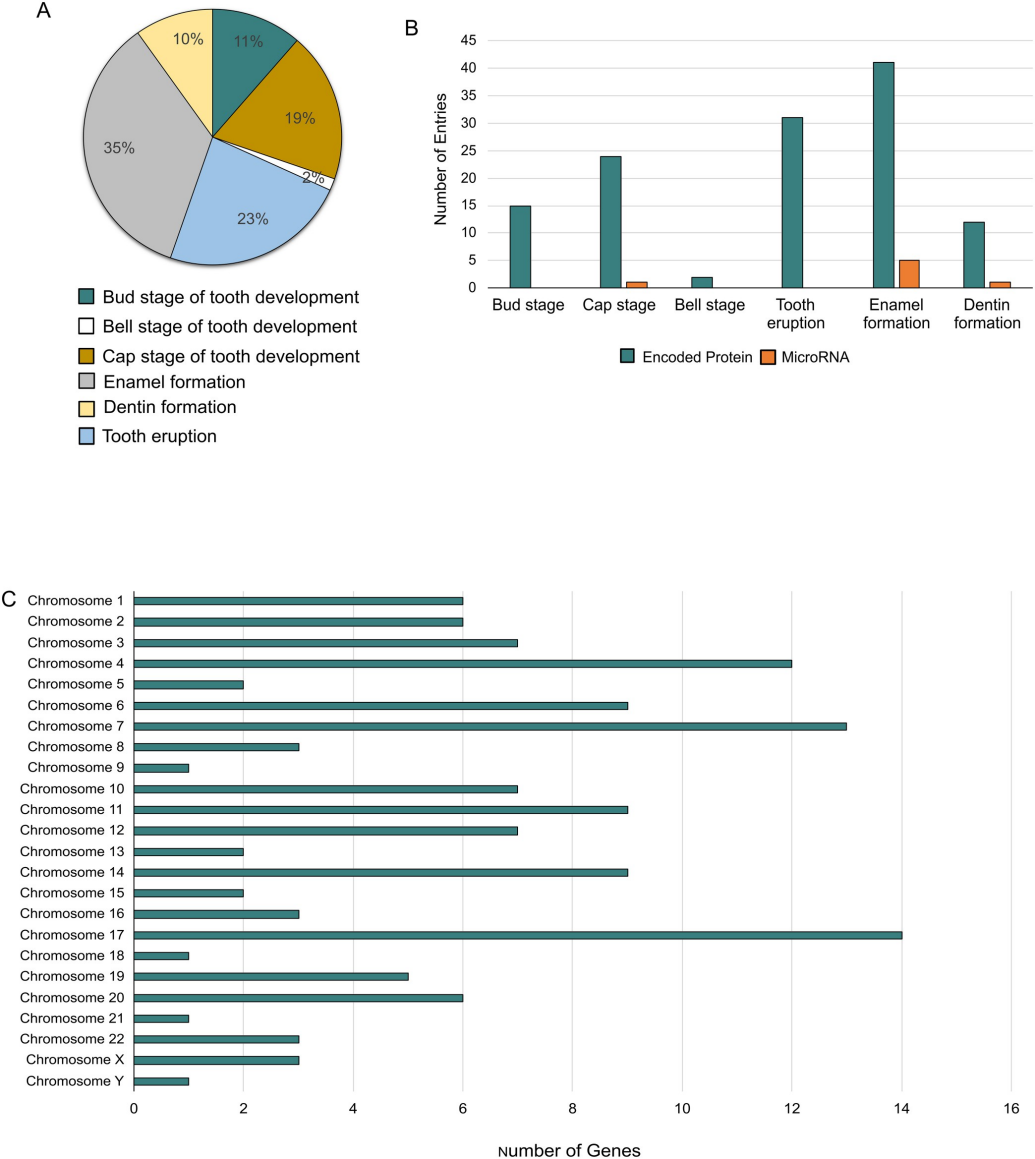
Distribution of genes (A) and, proteins and mRNAs (B) in different stages of tooth development. Chromosome-wide distribution of genes involved in tooth development.

Bioinformatics for Dentistry is freely available worldwide at https://dentalbioinformatics.com/. This database was created to compile genomic and proteomic data on tooth development under one platform to facilitate dental research and education. A systematic search was conducted to curate the available data. As of April 2024, Bioinformatics for Dentistry archives 132 genes involved in human tooth development. The majority (35%) of the included genes are involved in enamel formation and 23% in tooth eruption (Fig 3A). The genes encode 125 proteins and 7 microRNAs (Fig 3B). Most genes were located on chromosomes 17, 7, and 3 (Fig 3C). The dataset of this knowledgebase was also verified against NCBI and OMIM to ensure its credibility (Supporting information).

The genomic and proteomic data is rapidly expanding. Thus, maintaining the currency of the database is crucial. We aim to update the Bioinformatics for Dentistry knowledgebase annually by (i) including new content where needed, (ii) verifying the external links, and (iii) maintaining the database for its functionality and security.

### The web interface of the database

**Fig 4:**
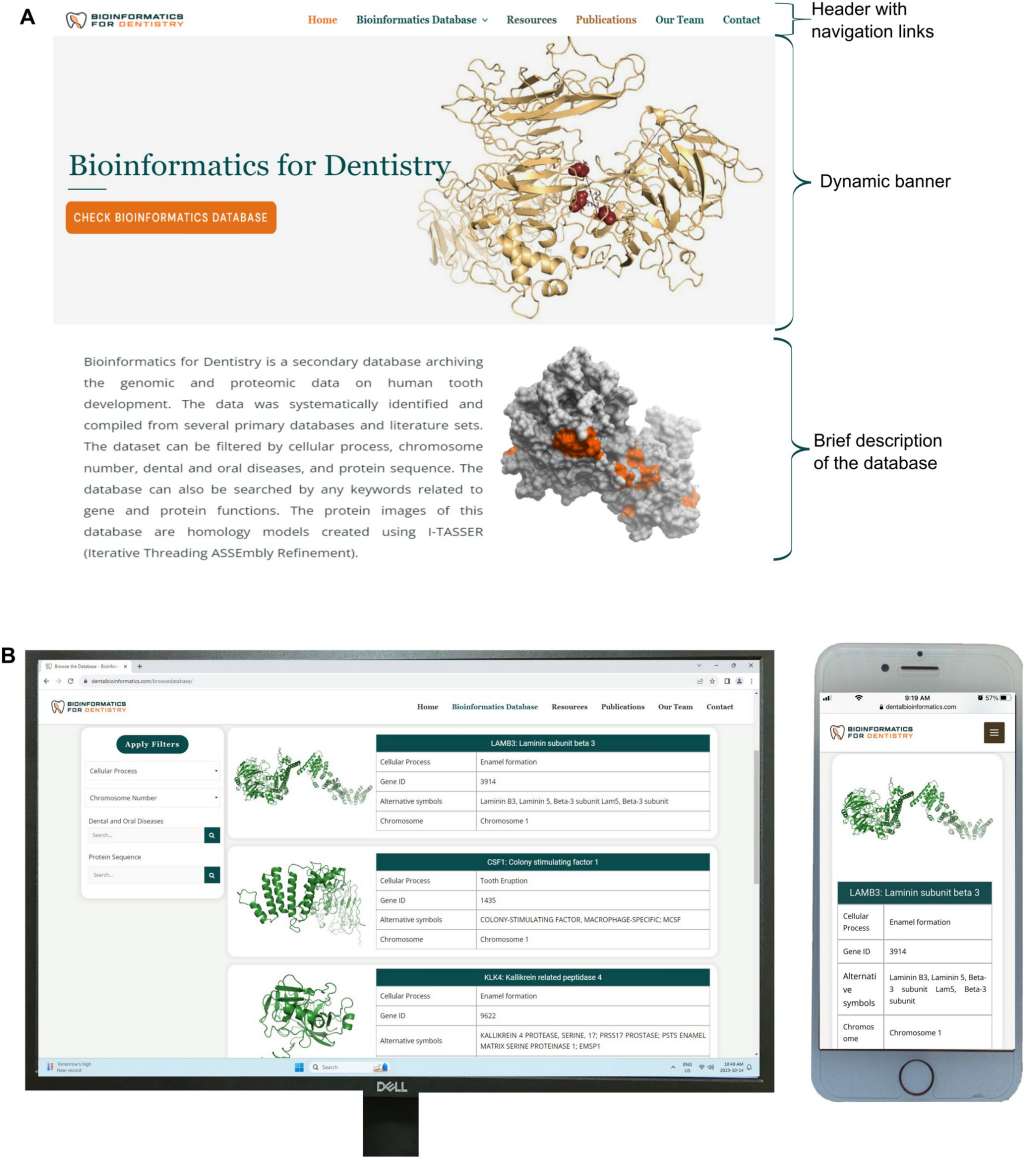
The web interface of Bioinformatics for Dentistry. The colors of the web interface were chosen for aesthetic harmony (A). The platform design is optimized to suit all types of displays and devices (B).

**Fig 5:**
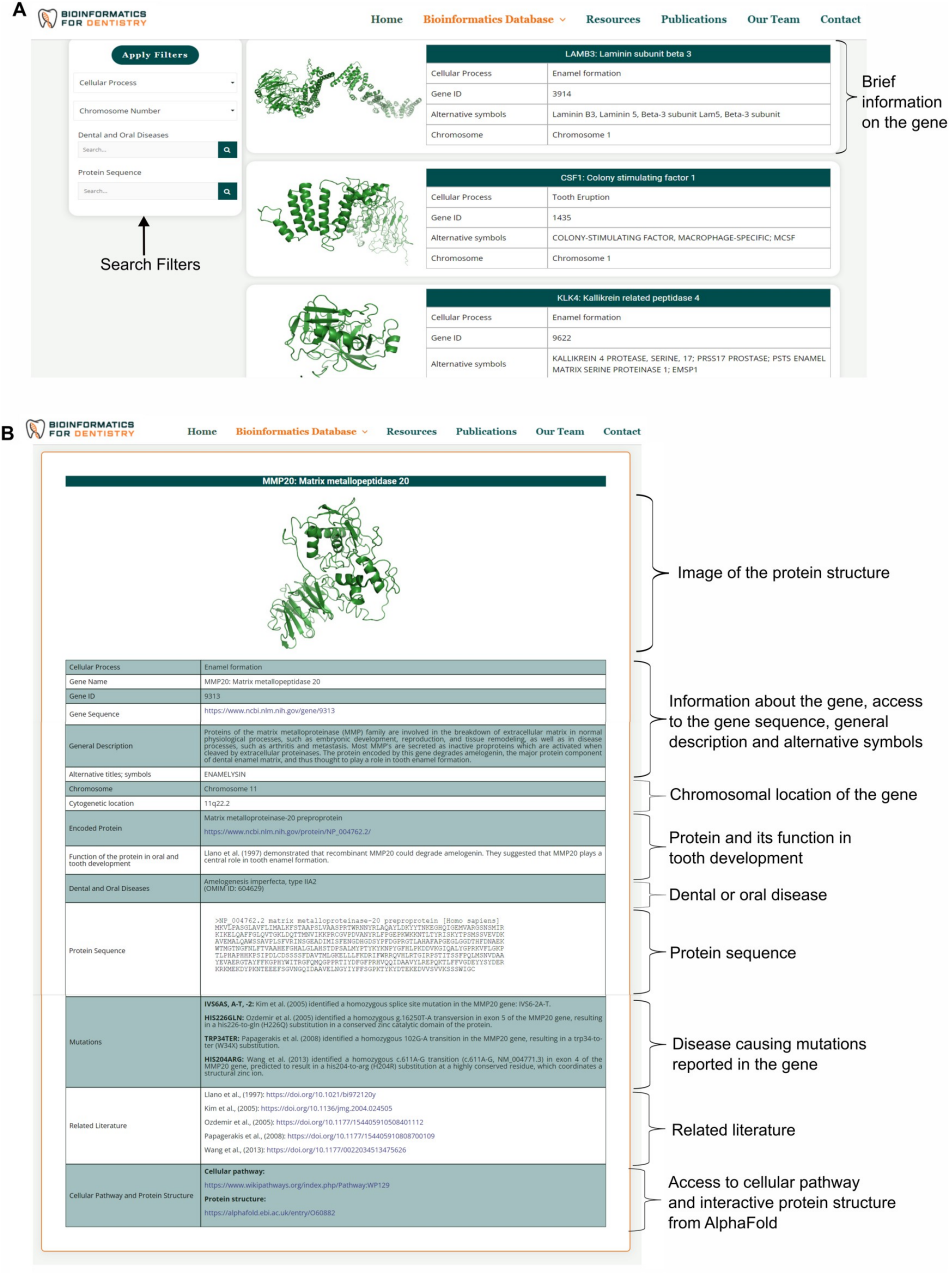
Data representation in the Bioinformatics for Dentistry. Browse the database page presents users with a list of genes displayed five genes per page, with brief information that includes cellular process, Gene ID, alternative symbols, and chromosome number (A). The detailed information page provides a large set of information related to the gene and its encoded product (B).

Bioinformatics for Dentistry offers a simple, easy-to-navigate, interactive platform for users to explore genes and proteins involved in human tooth development. It is indexed with Google and Bing to ensure worldwide accessibility. The colors of the tabs and background were chosen to match the logo and for aesthetic harmony. The platform design is optimized to suit a variety of different types of displays and devices. The homepage provides a dynamic, sliding banner, a brief database description, and links for navigation (Fig 4). The banner includes a large button directly linked to the database. The header includes additional links to allow users to browse the database, search the database, or explore more external resources (Fig 4A). As users navigate to browse the database, they are presented with a list of genes, displayed five genes per page, with brief information that includes cellular process, Gene ID, alternative symbols, and chromosome number (Fig 5A). By clicking on the gene name, users get access to the detailed information page specific to each gene (Fig 5B). The detailed information page provides information related to the gene and its encoded product, including protein name, sequence, and a description of its role in tooth development. Users can access the gene sequence from GenBank. Dental and oral diseases associated with the gene and the disease-causing mutation are also included. From the detailed information page, users can access the published literature, cellular pathways, and the predicted 3D structure of the protein (Fig 5B).

### Search and filters

**Fig 6:**
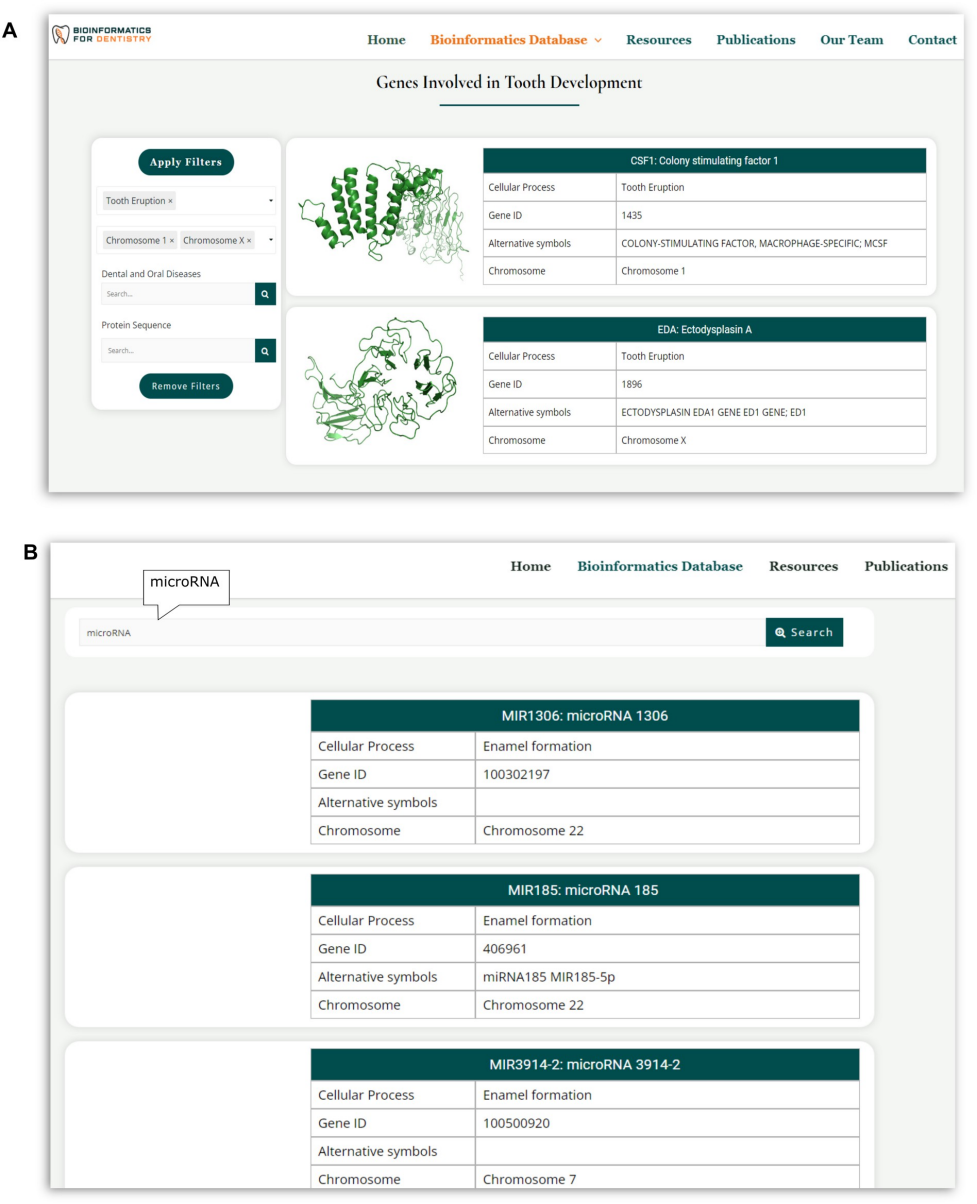
Search and Filter options in the Bioinformatics for Dentistry. (A)Four filters are available for users to conduct complex and specific searches. Multiple filters can be selected and applied to make the result more specific. This search result was returned by selecting ‘tooth eruption’ from the cellular process filter, and ‘chromosome 1’ and ‘chromosome X’ from chromosome number filter (B) Users can also search the database with any keywords to get the results.

Bioinformatics for Dentistry has two search options: (i) search using filters or (ii) search by keyword (Fig 6). The database contains embedded filters for complex and specific searches. The ‘Browse the Database’ link provides users access to the full database. The datasets are compiled in 27 pages; users can browse page by page or click ‘load more’ to display all data on the same page. Four filters are available on this page for users to use in any combination to identify specific data they need. Two filters, “cellular process” and “chromosome numbers” are selectable; users can select and activate these filters from a drop-down menu. “Dental and Oral Diseases” and “Protein Sequence” filters are text-based. Users can type a disease (e.g., dentinogenesis imperfecta) on the text box to find the results. They can also search the database for specific protein sequences. Multiple filters can be applied to make the search more targeted. For example, users can choose ‘tooth eruption’ from the cellular process filter to get the results of all 31 genes involved in tooth eruption. Adding additional filters, like ‘chromosome 1’ and ‘chromosome X,’ will trim the result and show only two genes, one located in chromosome 1 and the other in chromosome X, that are reported to be involved in human tooth eruption (Fig 6A).

Bioinformatics for Dentistry also offers free-text search options for simple, quick searches. The “search the database” link allows users to write free text and extract information related to the search from the whole dataset. For example, writing ‘microRNA’ in the search box returns seven microRNAs currently listed in the database to be involved in tooth development (Fig 6B).

### Gateway to analysis tools

To conduct further *in-silico* data analysis, Bioinformatics for Dentistry provides a gateway for users to large sets of primary databases, sequence analysis, and structure prediction tools currently available for free. Users can access these external websites through the links provided under the ‘Resources’ tab. From the ‘Resource’ page, users can access all the primary databases used in developing Bioinformatics for Dentistry. They can also access the Basic Local Alignment Search Tool (BLAST) to search for homologues and compare pair-wise and multiple sequence analysis using NCBI or Clustal Omega tools. Popular sequence analysis and structure prediction tools like ExPAsy and SWISS-MODEL are also available to users through our database. Notably, the science behind sequence alignment and sequence analysis tools is rapidly evolving, and a comprehensive knowledge of the strengths and limits of such bioinformatic tools is required to conduct meaningful sequence analysis.

## Discussion

Bioinformatics for Dentistry has been developed to benefit researchers, students, and oral health professionals involved in dental education and research. This curated knowledgebase compiles genomic and proteomic data related to human tooth development under one platform. Primary literature, supporting the role of a gene or protein in tooth development, is included for each entry of this database. Where studies on human models were not feasible, experiments reported in the primary literature were conducted on animal tissues. Although Bioinformatics for Dentistry is developed with a simple user interface, it is possible that users with limited bioinformatics expertise may find it challenging to extract meaningful insights from the dataset. As the database is freely available worldwide, it is possible that the effectiveness of its interface and search functionalities may vary depending on local restrictions. We acknowledge that despite our best efforts to curate scattered genetic data, it is possible to have missing information in the knowledgebase. It is essential to consider that the reliability and accuracy of the compiled data depend on the accuracy of the primary sources from which it was extracted. The inclusion criteria for this database may favor well-studied genes and proteins over those with limited research attention.

### Limitations

In its current phase, Bioinformatics for Dentistry has some limitations that we acknowledge and aim to overcome in the future. We do not have any built-in sequence analysis tool for users to conduct pair-wise or multiple sequence analysis. However, users are provided access to several external sequence analysis tools under the ‘Resources’ tab of this knowledgebase. Protein images from I-TASSER models are not available for all the proteins listed in our database in the current phase. As an alternative, users can access the interactive protein models from AlphaFold, which is available for all the proteins in the database. Gene sequence, cellular pathways, and protein structures are accessed from an external source, not embedded in our database. We acknowledge that while this approach allows for access to a wider range of data, it may introduce dependencies and potential inconsistencies if the external sources undergo changes or updates.

## Conclusion and future plans

Bioinformatics for Dentistry has been developed as a knowledgebase for researchers, students, and oral health professionals involved in dental education and research. To our knowledge, this is the only database compiling genomic and proteomic data related to human tooth development under one platform. This database can also be an excellent teaching tool for teaching genetics of tooth development in undergraduate dentistry programs. This database will be expanded in the future, incorporating more resources, like gene expression and protein interaction data related to tooth development. The database will contain built-in sequence analysis tools within the knowledgebase, enabling users to perform pair-wise or multiple sequence analysis directly on the platform. Using the built-in analysis tools, students and researchers will be able to conduct *in silico* studies, including the prediction of conserved protein domains, identification of the location, type, and functional impact of disease-causing mutations, and so on. Homology models will be created for all the proteins and their disease-causing mutants listed in the database using I-TASSER [26]. Mol* [27], an open-source toolkit, will also be incorporated to create an interactive platform for users to visualize and compare the wild-type and mutated protein structures. The interactive visual representations of protein structures can enhance users’ understanding of protein functions, enable them to compare wild-type and mutated protein structures, and thus further facilitate learning and research activities. The database will be updated once a year to incorporate new data and ensure functionality, security, and accessibility to external links. This work can assist in improving the understanding of the genetics of tooth development, stimulate new research initiatives, and lead to discoveries.

## Supporting information

S1 Table Content of the Bioinformatics for Dentistry, with its respective primary sources. Link: https://figshare.com/articles/dataset/S1_Table_docx/25546000.
(DOCX)

S2 Table List of genes and related information involved in tooth development. Link: https://figshare.com/articles/dataset/S2_Table/25546426.
(XLSX)

S1 Fig Verification of the data in Bioinformatics for Dentistry against NCBI and OMIM. Link: https://figshare.com/articles/figure/S1_Fig/25631880.
(PNG)
