# Supplementary material for: Bioinformatics for Dentistry: A secondary database for the genetics of tooth development
Source: PLoS One. 2024 Jun 6;19(6):e0303628. doi: 10.1371/journal.pone.0303628 (PMC11156362; doi:10.1371/journal.pone.0303628)
Supplement: S1 Table — Link: https://figshare.com/articles/dataset/S1_Table_docx/25546000. (DOCX) [file pone.0303628.s001.docx]

**Content of the Bioinformatics for Dentistry, with its respective primary sources**

| Content of the Database | Sources |
| --- | --- |
| *Genes involved in human tooth development*  Bioinformatics for Dentistry archives 132 human genes involved in tooth development. The genes are organized according to the stages of tooth development. For each gene entry, ‘Gene ID’ and link to the primary GenBank page is also included, providing access to the DNA sequence. | GenBank: National Library of Medicine (NCBI)  www.ncbi.nlm.nih.gov/gene |
| *Alternative symbol, chromosome number, and cytogenetic location*  For each of the 132 entries, all known alternative symbols, chromosome numbers, and specific cytogenetic locations are provided. | OMIM: Online Mendelian Inheritance in Man  www.omim.org/ |
| *General description of the gene*  This database section contains a brief general description of the gene and its encoded product. | GenBank: National Library of Medicine (NCBI)  www.ncbi.nlm.nih.gov/gene |
| *Proteins involved in human tooth development*  For each gene entry, information related to the encoded protein is incorporated. Link is provided for users to visit the source-page of the protein from NCBI. | Protein: National Library of Medicine (NCBI)  www.ncbi.nlm.nih.gov/protein/ |
| *Protein sequence*  The database archives the sequence of each protein in FASTA format. | Protein: National Library of Medicine (NCBI)  www.ncbi.nlm.nih.gov/protein/ |
| *Protein structure*  Knowledge of three-dimensional (3D) protein structure is essential for studying protein function. However, crystalized, complete protein structures related to teeth and oral development are rare. As an alternative to experimentally determined structure, users have access to computationally generated protein models from Bioinformatics for Dentistry database. For each protein listed in the database, uses have access to its interactive 3D protein structure generated from amino acid sequence using artificial intelligence.  For many proteins of the database, the users have additional access the static images of the homology models developed by the iterative threading assembly refinement (I-TASSER) server. | AlphaFold: Protein Structure Database  https://alphafold.ebi.ac.uk/  I-TASSER: Protein Structure & Function Predictions  https://zhanggroup.org/I-TASSER/ |
| *Function of the protein in oral and tooth development*  Users can learn about dental specific function of each protein included in the database. This section contains a brief description and experimental evidence confirming the role of the protein in tooth development. | OMIM: Online Mendelian Inheritance in Man www.omim.org/  PubMed https://pubmed.ncbi.nlm.nih.gov/ |
| *MicroRNA modulators of Tooth development*  Several genes involved in tooth development codes for microRNAs. Users of the database have access to the detail information of these microRNA modulators. | GenBank: National Library of Medicine (NCBI)  www.ncbi.nlm.nih.gov/gene |
| *Cellular Pathways*  For each protein listed in the database, users have access to cellular and metabolic pathways where the protein is known to play roles. | Reactome pathway knowledgebase  www.reactome.org/  WikiPathways  www.wikipathways.org/ |
| *Dental and Oral Disease*  Bioinformatics for Dentistry achieves the oral and dental diseases linked to the gene entries. Users can get the OMIM ID for each disorder, which will help them to study more from the primary source. | OMIM: Online Mendelian Inheritance in Man  www.omim.org/ |
| *Mutation*  For the gene entries, known mutations identified to cause dental and oral diseases are listed in the database. | OMIM: Online Mendelian Inheritance in Man  www.omim.org/ |
| *Literature*  Bioinformatics for Dentistry provides access to the primary literature related to protein function, dental disorders, and reported mutations. | PubMed https://pubmed.ncbi.nlm.nih.gov/ |
