# Supplementary figures and images for: Bioinformatics for Dentistry: A secondary database for the genetics of tooth development
Source: PLoS One. 2024 Jun 6;19(6):e0303628. doi: 10.1371/journal.pone.0303628 (PMC11156362; doi:10.1371/journal.pone.0303628)

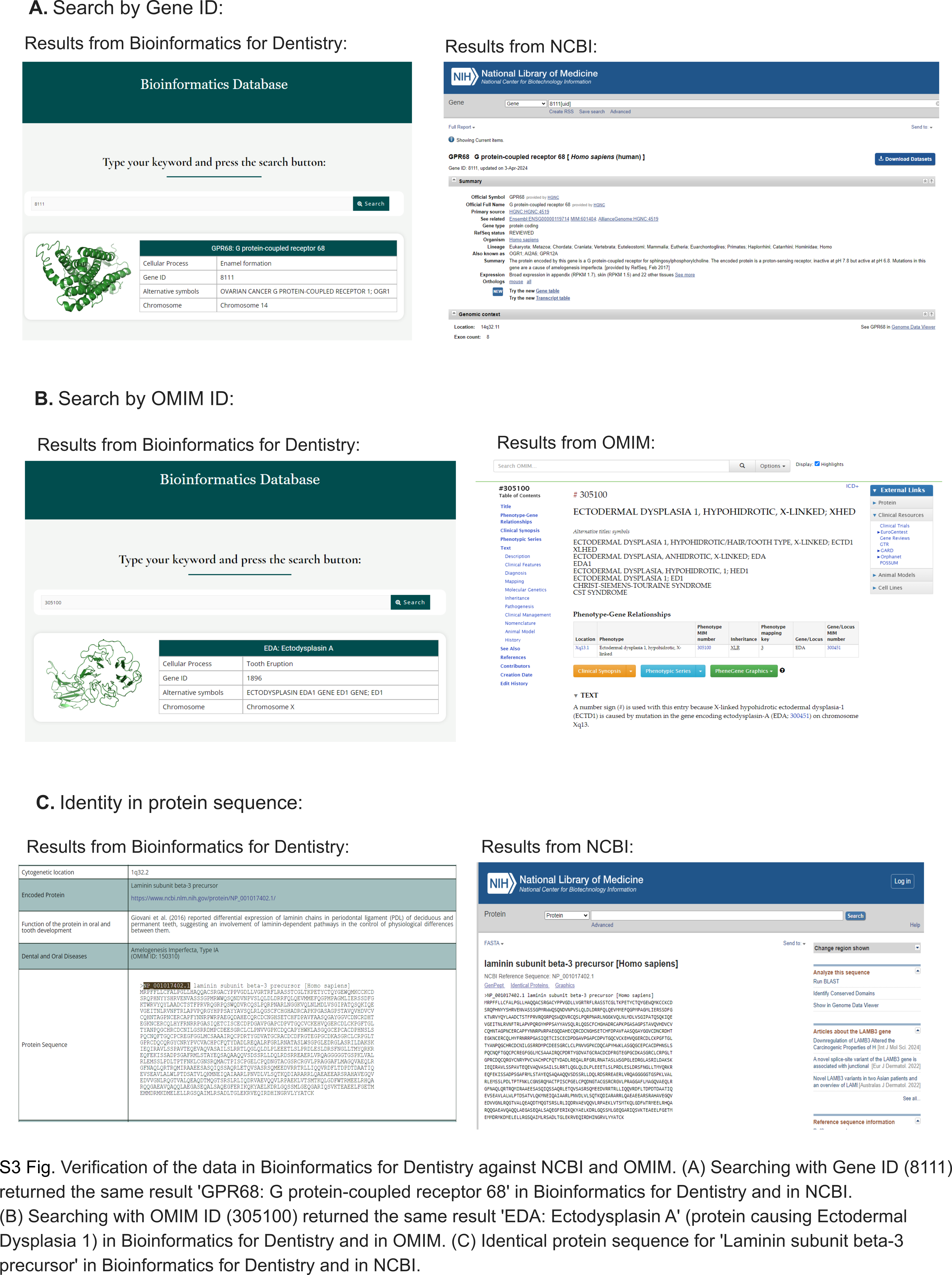

Supplement: S1 Fig — Link: https://figshare.com/articles/figure/S1_Fig/25631880. (PNG) [file pone.0303628.s003.png]
